# Role of Ezrin in Asthma-Related Airway Inflammation and Remodeling

**DOI:** 10.1155/2022/6255012

**Published:** 2022-12-07

**Authors:** Shumei Zhao, Jiaqi Luo, Jun Hu, Hesheng Wang, Ningwei Zhao, Meng Cao, Cong Zhang, Rongkui Hu, Lanying Liu

**Affiliations:** ^1^Affiliated Hospital of Nanjing University of Chinese Medicine, Jiangsu Province Hospital of Chinese Medicine, Nanjing 210029, China; ^2^Nanjing University of Chinese Medicine, Nanjing 210029, China; ^3^Shimadzu Biomedical Research Laboratory, Shanghai 200233, China; ^4^Affiliated Hospital of Integrated Traditional Chinese and Western Medicine Nanjing University of Chinese Medicine, Nanjing 210028, China

## Abstract

Ezrin is an actin binding protein connecting the cell membrane and the cytoskeleton, which is crucial to maintaining cell morphology, intercellular adhesion, and cytoskeleton remodeling. Asthma involves dysfunction of inflammatory cells, cytokines, and airway structural cells. Recent studies have shown that ezrin, whose function is affected by extensive phosphorylation and protein interactions, is closely associated with asthma, may be a therapeutic target for asthma treatment. In this review, we summarize studies on ezrin and discuss its role in asthma-related airway inflammation and remodeling.

## 1. Introduction

Global Initiative for Asthma (GINA) guidelines suggest that asthma is a serious global health problem, affecting patients of all age groups [1]. Its prevalence is increasing globally, with the number of affected patients estimated to increase to 400 million by the year 2025 [2]. It is a heterogeneous disorder characterized by chronic airway inflammation and remodeling [3–6]. Since asthma occurs with chronic airway inflammation, corticosteroids are the most widely prescribed anti-inflammatory agents for it [7]. Type 2 innate lymphoid cells (ILC2), type 2 T-helper (Th2) cells, and related cytokines (such as IL-4, IL-5, and IL-13) are major players in the pathological changes and clinical symptoms of asthma, and increasing attention has been paid to anti-IL-5, anti-IL-5R*α*, and anti-IL-4R*α* therapy [8]. However, asthma remains poorly controlled in about 53% of patients across Western Europe and Asia [9, 10], and patients with severe disease respond poorly to corticosteroids [11]. Moreover, about 30-60% of patients with refractory disease have fixed airway obstruction, attributed to airway remodeling, including increased smooth muscle mass, thickened reticular basement membrane, subepithelial collagen deposition, epithelial desquamation, goblet cell hyperplasia, and angiogenesis [3, 12, 13]. Airway remodeling might be a consequence of inflammation, or vice versa, therefore it is necessary to develop an approach to understanding the complex interplay between inflammation and airway structure.

Ezrin is a plasma membrane and cytoskeleton linking protein that is concentrated in actin-rich surface structures [14]. The plasma membrane-cytoskeleton complex is involved in physiological and pathological states of cell regulation, such as cell migration, cell proliferation, cell adhesion, and cytoskeletal remodeling [15]. Research demonstrates that ezrin may have a role in the pathogenesis of asthma by affecting both bronchial epithelial repair and contraction of airway smooth muscle cells. Moreover, it regulates the rho-associated protein kinase (*ROCK*), NF-*κ*B, and cyclic adenosine monophosphate/protein kinase A (cAMP/PKA) signal transduction pathways [16, 17]. In this review, we summarize studies on the structure, distribution, and regulation of ezrin, and discuss its role in airway inflammation and remodeling in asthma.

## 2. What Is Ezrin?

### 2.1. Gene and Structure

Ezrin is a principal member of the ezrin-radixin-moesin (ERM) family of proteins, and shares about 75% of the amino acid identity [18–20]. It was originally isolated from chicken intestinal brush border epithelium as a component of the microvilli in 1983 and named after Ezra Cornell, cofounder of Cornell University, where it was discovered [21–23]. Villin 2 (now called ezrin), the gene-encoding ezrin, is located on chromosome 6q25-6q26, contains 13 exons, is 24 kb long, and has an apparent molecular weight of 81 kDa [24, 25]. In 1988, ezrin was isolated from the microvillar membranes of human choriocarcinoma cells as a 75 kDa protein and named cytovillin 2 [26]. The difference in apparent molecular weight could be due to the structural changes caused by phosphorylation, which affects its electrophoretic mobility [16].

**Figure 1:**
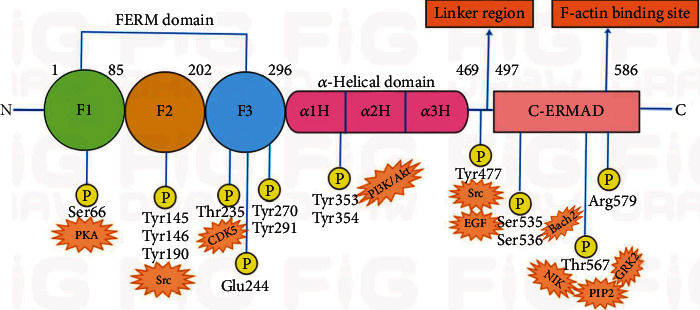
Schematic diagram of the ezrin protein domain. Ezrin protein has two functional domains connected through the *α*-helical domain: the N-terminal FERM (F for 4.1 protein, E for ezrin, R for radixin, and M for moesin, amino acid 1–296) domain and the C-terminal C-ERMAD (amino acid residues 470–586). The FERM domain comprises three subdomains (F1: a ubiquitin-like domain; F2: with four *α*-helices; and F3: a pleckstrin homology domain). C-ERMAD contains F-actin binding sites, which can bind to FERM domains or actin. The central helical (amino acid residues 297–469) domain comprises three *α* helices: *α*1H, *α*2H, and *α*3H, forming a coiled-coil in the monomer structure. Abbreviations: Thr: threonine; Tyr: tyrosine; Ser: serine; Arg: arginine; Glu: glutamic; PIP2: phosphatidylinositol 4,5-bisphosphate; NIK: Nck-interacting kinase; GRK2: G protein-coupled receptor kinase 2; CDK5: cyclin-dependent kinase 5; PI3K: phosphoinositide 3-kinase; Akt (PKB): protein kinase B; PKA: protein kinase A; EGF: epidermal growth factor.

Ezrin is the most studied member of the ERM family, and its structural composition has been highly conserved in the evolutionary process. The protein contains 586 amino acids and is mainly composed of three domains, the N-terminal FERM (F for 4.1 protein, E for ezrin, R for radixin, and M for moesin) domain, a central *α*-helical domain, and the C-terminal ERM association domain (C-ERMAD) [27–29]. The FERM domain is a 30 kDa composite domain (residues 1-296), which comprises three subdomains: F1, a ubiquitin-like domain; F2, with four *α*-helices; and F3, a pleckstrin homology domain [23]. These subdomains form a trilobed, cloverleaf structure with a rough resolution of 70 Å by 70 Å across and 40 Å deep [30–33]. The C-ERMAD domain (residues 470-586) contains the F-actin binding site that associates with the FERM domain of the N-terminal or actin [34, 35]. A central *α*-helical domain (residues 297-469) lies between the FERM domain and the C-ERMAD domain, which is composed of three helices: *α*1H, *α*2H, and *α*3H, forming a coiled-coil in the monomer structure [36]. A linker region is shown after the *α*-helical domain [37] (Figure 1).

Ezrin can have monomer and dimer states, with the former being a stable configuration that can function independently. It has an active open form localized at the plasma membrane and a dormant closed form localized in the cytoplasm [38]. In the dormant closed form, the coiled-coil in the *α*-helical domain folds back on itself and the C-ERMAD associates with the FERM domain of its own or other ERM family members to form monomers or homodimers/heterodimers, with actin and other membrane receptor binding sites wrapped inside [39, 40]. In this form, ezrin fails to cross-link the plasma membrane and the cytoskeleton. FERM is a labile domain [14], and ezrin is activated in the open conformation when phosphatidylinositol 4, 5-bisphosphate (PIP2) binds to the FERM domain, exposing C-ERMAD sites such as threonine and tyrosine for phosphorylation [15].

### 2.2. Expression and Subcellular Distribution

Ezrin is expressed in the lungs, intestines, stomach, and kidneys, and it exhibits clear tissue specificity [41]. It is mainly located in the epidermal rather than muscle cells [42, 43]. In the small intestine, ezrin expression is limited to the absorptive epithelium and the visceral mesothelium and is absent in the endothelium and smooth muscle [44]. It is mainly distributed on the apical aspect of the plasma membrane which is rich in actin, such as the microvilli, filopodia, ruffling membranes, and cell adhesion sites [24, 45]. Of note, ezrin remains inactive in the cytoplasm and exerts its key regulatory role in plasma membrane-based processes via direct interactions with other membrane proteins [46].

## 3. Posttranslational Modifications

### 3.1. Phosphorylation

Protein functions can be regulated by various posttranslational modifications (PTMs), like phosphorylation [47]. Ezrin is mainly subject to protein phosphorylation, which allows it to exert its crucial regulatory role. Ezrin is activated by recruitment to the plasma membrane by PIP2 and subsequent phosphorylation [48–50]. Thr567 in C-ERMAD is the most recognized target for phosphorylation-mediated ezrin activation [23]. It is phosphorylated by Nck-interacting kinase (*NIK*) and G protein-coupled receptor kinase 2 (*GRK2*), exposing the active sites through uncoupling of FERM and C-terminal domains. Thus, the transition of ezrin from inactive oligomers into active monomers occurs [51–53]. Phospho-deficient mutants, generated through replacement of Thr567 with alanine, exhibited increased apoptosis, demonstrating the necessity of Thr567 for ezrin activation [54]. Thr235 is phosphorylated by cyclin-dependent kinase 5 (*CDK5*) and cooperates with Thr576 for full activation of ezrin [23, 55] (Figure 1, Table 1).

Ezrin can undergo tyrosine (Tyr) phosphorylation through stimulation by platelet-derived and hepatocyte growth factors [41]. Tyr145 and Tyr146 can be phosphorylated by tyrosine specific Src kinases [56, 57]. Tyr190 may act as a docking point for the binding of Src Homology-2 (*SH2*) to ezrin, and that becomes the premise for phosphorylation of Tyr145 [24]. Tyr270 is on the *β* strand of the outer *β* sheet in subdomain F3, and its phosphorylation may affect FERM-membrane interaction [30]. Tyr291 phosphorylation might weaken FERM and C-terminal interaction and keep ezrin in an open state [30]. Tyr353 phosphorylation is regulated through the phosphoinositide 3-kinase (PI3K)/Akt pathway and mediated by CD81; leading to reorganization of the actin cytoskeleton and activation of B cells [58–60]. Tyr354 phosphorylation affects ezrin's link to mobility and cellular adhesion through the modulation of cellular tubulogenesis [57, 61]. Tyr477 phosphorylation promotes cell motility and invasion induced by Src kinase, which are required for the intrinsic Tyr kinase activity of growth factor receptors for epidermal growth factor (EGF) [62, 63] (Figure 1, Table 1).

Ser66 of subdomain F1, is a substrate for PKA, and its phosphorylation plays an important role in mediating remodeling of the apical membrane cytoskeleton associated with acid secretion in parietal cells [64]. Ser535 is essential for inhibiting the nuclear accumulation of Bach2 and its repressor activity in B cells, while Ser535 and Ser536 phosphorylation might weaken FERM and C-terminal interaction [30, 65] (Figure 1, Table 1).

### 3.2. Dephosphorylation and Other PTMs

Ezrin is also regulated through dephosphorylation and hydrolysis of PIP2. Dephosphorylation is the process of removing phosphate groups from an organic compound (such as ATP) by hydrolysis. During cell division, a kinetochore-localized PP1-sds22 complex induces dephosphorylation and inactivation of polar ezrin [66]. The dephosphorylation of ezrin induced by T-cell receptor is related to the redistribution of CD43 and the formation of an immunological synapse. Activation of stromal cell-derived factor-1 (SDF-1) and its downstream phospholipase C (PLC) rapidly reduced PIP2 levels and induced ezrin dephosphorylation and disassociation from the cell membrane in lymphocytes. [67–69] Dephosphorylation of ezrin by protein tyrosine phosphatase (PTP*σ*), protein phosphatase 2A (PP2A), and protein phosphatase 2C (PP2C) has been demonstrated through *in vitro* studies [41, 70]. Arg579 and Glu244 form a tight salt bridge, which is important for the affinity between FERM and the C-terminal [30, 71]. Lys60 acetylation has also been reported, and it may impair the phosphorylation-dependent interactions of ezrin [72] (Figure 1, Table 1).

## 4. Protein Interactions

### 4.1. Interaction of Ezrin with PIP2

PIP2 constitutes only 1% of the total cellular acidic lipid; however, it is the most abundant phosphoinositide that recruits ezrin to the plasma membrane [73–75]. A binding site, specific for PIP2 that uncouples FERM from the C-terminal plays an essential role in the activation of ezrin [76–78]. The lysines located in subdomains F1 and F3 have a strong effect on the binding of ezrin to PIP2 [14, 73]. Priming of ezrin for phosphorylation requires an appropriate charge distribution provided by phosphate groups in the head group of PIP2, and a high local concentration of the head group can only be achieved in vitro using micelles or liposomes. Moreover, the central *α*-helical coiled-coil hairpin is necessary for the priming of ezrin for phosphorylation, which counteracts the strong association between FERM and C-ERMAD. Upon binding with PIP2, this effect was enhanced [79]. Additionally, PIP2 binding increased the number of weak bonds between ezrin and F-actin, thereby upregulating the adhesion energy and membrane tension to regulate cell adhesion, migration, and morphology [80].

### 4.2. Interaction of Ezrin with Other Proteins

Several proteins can directly or indirectly interact with the FERM domain. PIP2 binds to cytoplasmic tails of intracellular adhesion molecule-1, -2 (ICAM-1, -2), inducing an interaction between ICAM-1, -2 and ezrin, thus participating in the phosphoinositide signaling pathways [81]. Meanwhile, a complex between the cytoplasmic tail of CD44 and FERM or the entire length of ezrin forms, and coordinates the spatial and temporal localization of PIP2-CD44-ezrin complex-mediated signaling events [82]. CD43 regulates T-cell trafficking through Ser76 in its cytoplasmic tail, while its binding to ezrin is crucial for Ser76 phosphorylation [83]. Ezrin regulates E-cadherin-dependent adherens junction assembly by activating the GTPase Rac1 rather than RhoA or Cdc42 [84]. The Na^+^/H^+^ exchanger 1 (NHE1) activates and recruits ezrin to specific plasma membrane, thus regulating cell survival as a scaffold for the recruitment of a signal complex that consists of PI3K and Akt [85, 86]. Ezrin can anchor to the plasma membrane using postsynaptic density proteins (PDZs), such as the Na^+^-H^+^ exchanger regulatory factor (NHERF1), also known as the ERM-binding phosphoprotein 50 (EBP50), and regulatory proteins such as NHE3 kinase (E3KARP, also known as NHERF2) [23, 87]. PKA is linked physically and functionally to cystic fibrosis transmembrane conductance regulator (CFTR) by an A-kinase anchoring protein (AKAP) interaction, suggesting that ezrin serves as an AKAP for PKA-mediated phosphorylation of CFTR [88], and as the specific binding site, ezrin binds to PKA at the *α*-helical domain (amino acids 363–470) [89, 90]. (Table 2).

## 5. Role of Ezrin in Asthma-Related Airway Inflammation and Remodeling

### 5.1. Role of Ezrin in Asthma-Related Airway Inflammation

**Figure 2:**
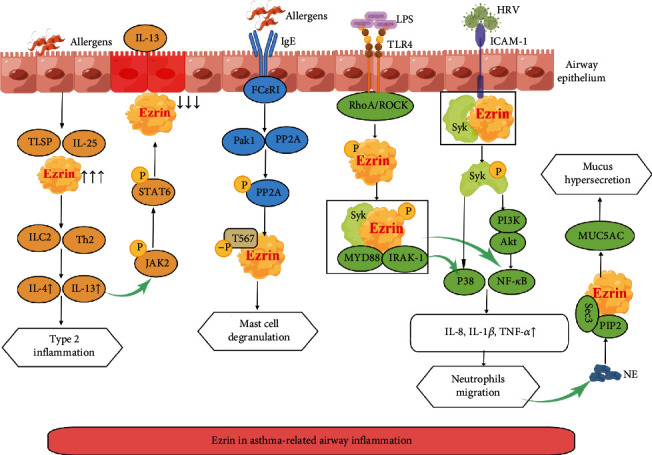
Role of ezrin in asthma-related airway inflammation. Abbreviations: ILC2: type 2 innate lymphoid cells; Th2: type 2 T-helper cells; P: phosphorylate; -P: dephosphorylate; JAK2: Janus Kinase 2; STAT6: signal transducers and activators of transcription 6; TSLP: thymic stromal lymphopoietin; Fc*ε*RI: Receptor I for the Fc Fragment of IgE; Pak1: kinase 1; PP2A: protein phosphatase 2A; T567: threonine 567; LPS: Lipopolysaccharide; TLR4: toll-like receptor 4; ROCK: Rho-associated coiled-coil containing protein kinase; MyD88: myeloid differentiation factor 88; IRAK-1: IL-1R-associated kinase 1; HRV: human rhinovirus; ICAM-1: intracellular adhesion molecules-1; Syk: spleen tyrosine kinase; PI3K: phosphoinositide 3-kinase; Akt (PKB): protein kinase B; NE: Neutrophil elastase; PIP2: phosphatidylinositol 4,5-bisphosphate; MUC5AC: robust mucin 5 AC; IL-25: interleukin-25; IL-4: interleukin-4; IL-13: interleukin-13; IL-8: interleukin-8; IL-1*β*: interleukin-1*β*; TNF-*α*: tumor necrosis factor-*α*.

As is known to all, the pathological mechanism of asthma is type 2 airway inflammation involving type 2 innate lymphoid cells (ILC2) and type 2 T-helper (Th2) cells. Allergens are the main triggers [91]. Airway epithelial cells are the first line of defense, releasing alarmins interleukin-25 (IL-25), thymic stromal lymphopoietin (TSLP), ezrin, and initiating the immune cascade, leading to a release of interleukin-4 (IL-4) and interleukin-13 (IL-13) [92]. IL-4 and IL-13 are called prototypical type 2 cytokines, which can cause immunoglobulin E (IgE) production and eosinophils can transport to the airways [91], thus triggering an asthma attacks. Furthermore, IL-13 induces the phosphorylation of Janus Kinase 2 (JAK2), followed by increased nuclear translocation of phosphorylated signal transducers and activators of transcription 6 (STAT6), leading to downregulated ezrin expression, which is related to IL-13-induced epithelial injury [93]. Ezrin may be a potential biomarker of epithelial damage in asthma (Figure 2).

Mast cells are crucial in asthma; they secrete various proinflammatory mediators [94]. IgE-mediated crosslinking of the high-affinity receptor for IgE (Fc*ε*RI) with allergens induces the p21-activated kinase 1 (Pak1) binding and activation to PP2A. PP2A then dephosphorylates ezrin at threonine 567 to promote the degranulation of mast cells [95], leading to the asthmatic response (Figure 2).

Patients with asthma are usually affected by exacerbations resulting from nasal pathogens. The airway epithelium is the primary target of inhaled pathogens like human rhinovirus (HRV), a common trigger of acute exacerbations of asthma. It has been demonstrated that ezrin forms a protein complex with ICAM-1 and spleen tyrosine kinase (Syk) at the plasma membrane and subsequently activates Syk for HRV internalization. Meanwhile, the phosphorylation of Syk induces the activation of p38 mitogen-activated protein kinase (MAPK) and PI3K and interleukin-8 (IL-8) expression [46, 96–98]. In addition, lipopolysaccharide (LPS) acts on toll-like receptor 4 (TLR4), rapidly activating Rho-associated coiled-coil-containing protein kinase (ROCK) to phosphorylate ezrin, and then ezrin transfers from the cytoplasm to the plasma membrane [99–101]. With the assistance of Syk and myeloid differentiation factor 88 (MyD88), ezrin interacts with IL-1R-associated kinase 1 (IRAK-1) to activate the NF-*κ*B and p38 pathways, promoting the expression of inflammatory factors tumor necrosis factor-*α* (TNF-*α*), and interleukin-1*β* (IL-1*β*) [101]. IL-8, IL-1*β*, and TNF-*α* induce chemotaxis and migration of neutrophils to the site of inflammation, causing airway inflammation in asthma [102–104]. Another dimension is that after stimulating neutrophil elastase (NE) generated by neutrophils, ezrin, and Sec3 are recruited to the cytoplasmic membrane, and with the participation of PIP2, induces robust mucin 5 AC (MUC5AC) protein secretion [105], leading to increased mucus secretion, which further aggravates asthma (Figure 2).

As an anti-inflammatory mediator, IL-10 is expressed by B cells and plays a role in suppressing the immune response of asthma [106]. The research shows that the genetic deletion of ezrin in B cells amplifies B-cell receptor (BCR) signaling, and ezrin phosphorylation limits the production of IL-10 in B cells [107]. Interestingly, ezrin peptide therapy involving human ezrin peptide one (HEP1) and regulatory ezrin peptide glycine 3 (RepG3) may switch the “receptor” form of ezrin to the submembrane active form, organizing cell signaling complexes and quickly engaging a cell-surface receptor, to enhance B cell activation and IL-10 production and suppress inflammation [108]. Hence the regulation of ezrin-multiprotein complexes is rather complicated.

The severity of asthma is closely related to airway inflammation; thus, reducing airway inflammation is vital in asthma treatment. Ezrin is overexpressed in the bronchi of humans with chronic airway diseases [105], involving ILC2, Th2, mast cells, neutrophils, B cells, IL-4, IL-13, IL-8, IL-1*β*, TNF-*α*, and other cytokines. All these findings will contribute to finding new targets for asthma treatment.

### 5.2. Role of Ezrin in Asthma-Related Airway Remodeling

Airway remodeling, based on airway inflammation, is one of the pathological characteristics of asthma, including epithelial damage, goblet cell hyperplasia, abnormal deposition of extracellular matrix (ECM), airway smooth muscle (ASM) cell proliferation, fibroblast activation, and angiogenesis [3, 109].

#### 5.2.1. Ezrin and Epithelial Integrity

**Figure 3:**
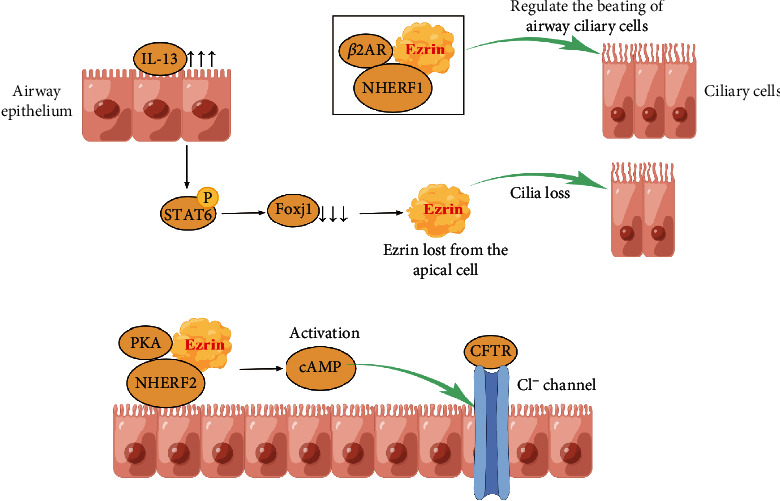
Ezrin and epithelial integrity. Abbreviations: *β*2AR: *β*2 adrenergic receptor; NHERF1: Na + −H+ exchanger regulatory factor; IL-13: interleukin-13; P: phosphorylate; STAT6: signal transducers and activators of transcription 6; Foxj1: forkhead box protein J1; PKA: protein kinase A; NHERF2: NHE3 kinase; cAMP: cyclic adenosine monophosphate; CFTR: cystic fibrosis transmembrane conductance regulator.

The major defense mechanism of the airway epithelium is mucociliary clearance conducted by airway ciliary cells cooperating with the protective mucous layer [110]. Ezrin via NHERF1 regulates the beating of airway ciliary cells by promoting the apical surface localization of *β*2 adrenergic receptor (*β*2AR) [110]. IL-13 directly acts on airway epithelial cells, interferes with the apical localization of ezrin, affects the differentiation of ciliary cells, reduces the ciliary beat frequency of ciliated epithelial cells, and increases mucus production [111]. Research also suggests that forkhead box protein J1 (foxj1) is required for apical localization of ezrin in airway epithelial cells [112]. IL-13 phosphorylates STAT-6 and transfers it to the nucleus to bind the foxj1 promoter to inhibit the expression of foxj1. Ezrin was lost from the apical cell compartment, leading to cilia loss and goblet cell hyperplasia [113]. Besides, in airway epithelial cells, the Ezrin-E3KARP-PKA complex stimulates CFTR Cl- channels through cAMP to regulate water and salt balance [114–117]. Clearly, ezrin plays an important role in maintaining epithelial integrity (Figure 3).

#### 5.2.2. Ezrin and Epithelial-Mesenchymal Transition (EMT)

EMT refers to the phenotypic change from epithelial to mesenchymal morphology, a key step of airway remodeling in asthma [118]. Among them, collagen I, *α*-smooth muscle actin (*α*-SMA), and fibronectin are mesenchymal markers [119]. Transforming growth factor-*β*1 (TGF-*β*1) and epidermal growth factor (EGF) are well-known inducers of EMT [120]. TGF-*β*1 binds to its receptor, increasing collagen I via the Smad-dependent pathway. Ezrin is required for TGF-*β*1-induced EMT in airway epithelial cells [121]. TGF-*β*1 also induces the expression of CD44, and ezrin activated by ROCK forms a complex, leading to EMT induction in alveolar epithelial cells [122]. Another inducer EGF induces phosphorylation of Ezrin Tyr353 and Thr567, causing EMT. It may be related to the NF-*κ*B pathway [123, 124].

#### 5.2.3. Ezrin, ASM Cells, and Fibroblasts

ASM cells and fibroblasts are key cells driving airway remodeling in asthma [3]. The ASM cells are considered to be part of the contractile or relaxant apparatus of the airways [125]. As an AKAP, ezrin combines with EBP50 to form a complex with PKA, activates cAMP signaling in ASM cells, and regulates the contraction and relaxation of ASM cells. This signaling can also drive CCAAT-enhancer-binding protein *β* (c/EBP*β*) to cause IL-6 gene expression and cytokine production, thus promoting the inflammatory process and airway remodeling [126, 127]. Fibroblasts are the major producer of ECM in asthmatic airways [3]. For one thing, ezrin may plays role in maintenance of fibroblast size and mechanical force by organizing actin filaments and regulating cortical stiffness. For another, ezrin promotes the proliferation of fibroblasts and collagen deposition through YAP (the cascade effector of the Hippo signaling pathway), causing ECM and fibrosis [128, 129].

## 6. Discussion

Increasing attention has been paid to ezrin in asthma pathophysiology. We summarized that ezrin regulates airway inflammation through type 2 immune cells, mast cells, neutrophils, and B cells and participates in airway remodeling via epithelial cells, EMT, ASM cells, and fibroblasts in asthma. Ezrin has various phosphorylation sites and binding partners and plays pivotal roles depending mainly on the form of the multi-protein complex. However, the related biological and clinical significance requires more study to explore its role as a potential biomarker and therapeutic target in asthma treatment.

## Figures and Tables

**Table 1:** Posttranslational modifications of ezrin protein.

Phosphorylation site	Kinases/phosphorylation factors	Biological effects	References
Serine-66	Protein kinase A (PKA)	Mediate the remodeling of the apical membrane cytoskeleton	[64]
Tyrosine-145, 146	Src	Participate in cell proliferation, adhesion, and migration	[56, 57]
Tyrosine-190	Src Homology-2 (SH2)	A docking point, the premise for phosphorylation of Tyrosine-145	[24]
Threonine-235	Cyclin-dependent kinase 5 (CDK5)	Cooperates with Threonine-576	[23, 55]
Tyrosine-270	—	Affect the FERM-membrane interaction	[30]
Tyrosine-291	—	Weaken the FERM and C-terminal interaction	[30]
Tyrosine-353	Phosphoinositide 3-kinase (PI3-kinase) PI3-kinase/Akt pathway	Participate in the reorganization of the actin cytoskeleton	[58–60]
Tyrosine-354	—	Link to mobility and cellular adhesion	[57, 61]
Tyrosine-477	Src, epidermal growth factor (EGF)	Promotes the cell diffusion	[62, 63]
Serine-535, 536	Bach2	Weaken the FERM and C-terminal interaction	[30, 65]
Threonine-567	Phosphatidylinositol 4,5-bisphosphate (PIP2) Nck-interacting kinase (NIK) G protein-coupled receptor kinase 2 (GRK2)	Uncoupling of FERM and C-terminal domains	[51–53]
Arginine579 Glu244	—	Form a tight salt bridge	[30]

**Table 2:** Regulation of ezrin by other proteins.

Proteins	Biological effects	References
Intracellular adhesion molecules-1 (ICAM-1) Intracellular adhesion molecules-1 (ICAM-2)	PIP2 binds to cytoplasmic tails of ICAM-1, -2, forming ezrin-multiprotein-complexes	[81]
CD44	The cytoplasmic tail of CD44 forms a complex with FERM or with the full-length ezrin	[82]
CD43	Regulates T-cell trafficking through Ser76 within its cytoplasmic tail	[83]
E-cadherin	Activate GTPase Rac1	[84]
Na+/H+ exchanger 1 (NHE1)	Activate and target ezrin to specific plasma membrane domains, regulate cell survival	[85, 86]
Na + −H+ exchanger regulatory factor (NHERF1)/ERM-binding phosphoprotein 50 (EBP50) NHE3 kinase (E3KARP)/NHERF2	Anchor to the plasma membrane using postsynaptic density proteins	[23, 87]
Protein kinase A (PKA) Cystic fibrosis transmembrane conductance regulator (CFTR)	PKA associates with CFTR via interaction with ezrin	[88]
